# Pulmonary Vein Stenosis: A Rare Disease with a Global Reach

**DOI:** 10.3390/children8030198

**Published:** 2021-03-06

**Authors:** Jennifer Schramm, Sivakumar Sivalingam, Guillermo E. Moreno, Dinh Quang Le Thanh, Kimberlee Gauvreau, Kaitlin Doherty-Schmeck, Kathy J. Jenkins

**Affiliations:** 1 Department of Cardiology, Children’s National Hospital, Washington, DC 20010, USA; jschramm2@childrensnational.org; 2 Department of Cardiothoracic Surgery, National Heart Institute, 50400 Kuala Lumpur, Malaysia; sivakumar@ijn.com.my; 3 Department of Cardiac Intensive Care, Hospital de Pediatría “Professor Dr. Juan P. Garrahan”, 412-6000 Ciudad de Buenos Aires, Argentina; guillermo.moreno1@gmail.com; 4 Department of Cardiac Surgery, Children’s Hospital 1, 700000 Ho Chi Minh City, Vietnam; dql.thanh@gmail.com; 5 Center for Applied Pediatric Quality Analytics, Department of Cardiology, Boston Children’s Hospital, Boston, MA 02115, USA; kimberlee.gauvreau@cardio.chboston.org (K.G.); kaitlin.doherty-schmeck@childrens.harvard.edu (K.D.-S.)

**Keywords:** pulmonary vein stenosis, low-middle income countries, congenital heart surgery

## Abstract

Pulmonary vein stenosis (PVS) is a rare, but high mortality and resource intensive disease caused by mechanical obstruction or intraluminal myofibroproliferation, which can be post-surgical or idiopathic. There are increasing options for management including medications, cardiac catheterization procedures, and surgery. We queried the International Quality Improvement Collaborative for Congenital Heart Disease (IQIC) database for cases of PVS and described the cohort including additional congenital lesions and surgeries as well as infectious and mortality outcomes. IQIC is a quality improvement project in low-middle-income countries with the goal of reducing mortality after congenital heart surgery. Three cases were described in detail with relevant images. We identified 57 cases of PVS surgery, with similar mortality to higher income countries. PVS should be recognized as a global disease. More research and collaboration are needed to understand the disease, treatments, and outcomes, and to devise treatment approaches for low resource environments.

## 1. Introduction

Pulmonary vein stenosis (PVS) is a rare, but frequently fatal disease caused by physical obstruction or intraluminal myofibroproliferation, either post-surgical or idiopathic [1]. Intraluminal PVS is frequently associated with total anomalous pulmonary venous return (TAPVR) repair, but can be related to prematurity or be idiopathic [2]. Intraluminal PVS is the subject of much research and is known to be partially related to neoproliferation of myofibroblast-like cells in the neointima though the inciting cause is not known [1,3,4,5,6,7]. Antiproliferative therapy, catheter-based interventions, and surgery continues to evolve in high income countries with increasing success, but the need for re-intervention is high [2,8]. The disease burden for PVS, and even TAPVR, in low-and-middle income countries (LMIC) is unknown. However, it is known that the rates of congenital heart disease (CHD) and deaths associated with CHD have increased in LMIC [9]. Increasingly, LMIC are performing complex repairs of TAPVR and managing idiopathic forms of PVS necessitating an understanding of the disease burden.

The International Quality Improvement Collaborative for Congenital Heart Surgery: Improving Care in Low-and-Middle Income Countries (IQIC) was started in 2008 with the mission of reducing mortality and post-operative infections using quality improvement techniques. The IQIC collects data regarding surgical procedures from participating sites, including patient demographics, surgical procedure details, and post-operative outcome, including mortality. We retrospectively reviewed the IQIC database to identify cases of PVS surgery from participating sites to describe the cohort and report the infectious and mortality outcomes.

## 2. Materials and Methods

Programs in LMIC participate in IQIC on a voluntary basis and submit deidentified data to a REDCap registry (REDCap Cloud, Encinitas, CA, USA). Sites and data underwent audits as previously published [10,11,12,13]. Approval to conduct research using IQIC data was obtained from Boston Children’s Hospital Institutional Review Board and local participation agreements in accordance with local standards. Cases were included for analysis if the surgical date was between 2012 and 2019 and if PVS was listed as a diagnosis. All additional diagnoses and ages were included as were children who had previous surgical procedures prior to PVS repair. Cases were excluded if PVS was listed, but there was no evidence of PVS in the remainder of the record (i.e., listed as PVS when pulmonary valve stenosis was indicated elsewhere). Demographics including country, age, gender, gestational age, hospital course information, and mortality were collected. Categorical variables are summarized with frequencies and percentages, and continuous variables with medians and 25th and 75th percentiles (interquartile range, IQR).

## 3. Results

**Figure 1:**
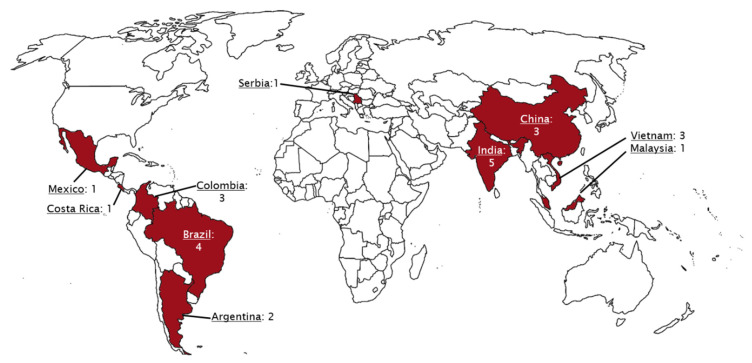
World map with countries highlighted. The numbers reflect the number of institutions reporting cases in the respective country. Cases spanned the globe with a slight predominance for countries in Asia. Note no cases were reported in Africa.

**Figure 2:**
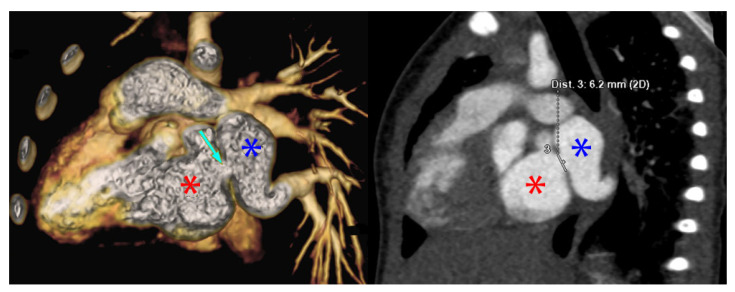
CT scans from the above described patient from Malaysia with repaired supracardiac TAPVR with recurrent PVS. The image on the left is a 3D reconstruction of a CT angiogram. The blue star denotes the pulmonary vein confluence, the red start denotes the left atrium. The light blue arrow on the left image denotes the area of stenosis. The image on the right that of the traditional CT angiogram with blue and red stars denoting the pulmonary veins and left atrium, respectively. The line measures the stenosis at 6.2 mm. Cross sectional imaging such as this allows institutions to risk stratify cases and offer surgery to those patients with the best predicted outcome.

**Figure 3:**
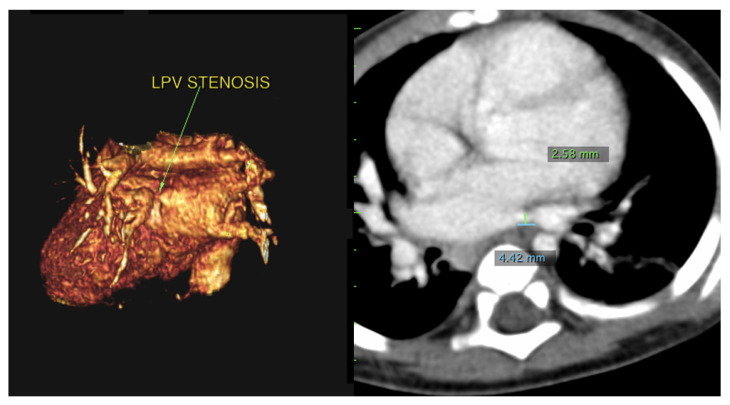
CT scans from the above described patient from Vietnam with left common pulmonary vein stenosis. The left image is a 3D reconstruction of a CT angiogram with the view of the left pulmonary vein connection to the left atrium. The arrow denotes the area of stenosis. The right image is a traditional CT angiogram with sternum anterior and spine posterior. The green marker is of the stenosis which narrows to 2.58 mm. The blue marker is of the length of the area of stenosis which is 4.42 mm.

Using PVS as an initial search term, 57 cases from 24 different institutions were identified and included for analysis. Table 1 is a list of the institutions reporting cases of PVS and Figure 1 is a map of this. Figure 2 and Figure 3 are of a computed tomographic (CT) scans performed on described cases from Malaysia (Figure 2) showing repaired TAPVR with recurrent stenosis and Vietnam (Figure 3) showing idiopathic common left pulmonary vein stenosis.

### Case Descriptions

“The patient was a 1 month old, ex-33 week gestation, child with failure to thrive. She was diagnosed with supracardiac TAPVR which was obstructed at the entry into the vertical vein. She underwent urgent repair of at 1 month of age at another tertiary care hospital. 3 months following surgery, an echocardiogram revealed mild to moderate pulmonary vein stenosis at the pulmonary anastomotic site for which she was transferred to the National Heart Institute. A CT revealed all pulmonary veins draining into the left atrium with severe stenosis at the pulmonary vein confluence to left atrium anastomosis. A re-operation was performed to relieve the pulmonary vein stenosis 9 months following the initial surgery using the sutureless technique. On discharge the echocardiogram showed a wide open pulmonary venous confluence with no gradient across the anastomotic site (Figure 2).”

—Sivakumar Sivalingam, FRCS(C.Th), Clinical Director Congenital Heart Surgery, Kuala Lumpur, Malaysia

“The patient was a 9-month-old who presented due to cough and shortness of breath. An echocardiogram revealed a small perimembranous ventricular septal defect (VSD) that was not hemodynamically significant and routine follow up was recommended. She continued to have respiratory difficulty at home and re-presented. A repeat echocardiogram re-demonstrated the VSD and showed left pulmonary vein stenosis. She was admitted for a CT which showed 2 left pulmonary veins to a common left pulmonary vein with stenosis at the common vein to left atrial connection. Right pulmonary veins were normal. She underwent VSD closure and sutureless anastomosis for left pulmonary veins 2 months later. The repair was delayed because of other urgent cases at the institution (Figure 3).”

—Dinh Quang Le Thanh, MD, MS, Pediatric General and Cardiac Surgeon, Children’s Hospital 1, Ho Chi Minh City, Vietnam

“The patient was an ex-full term 2-month-old who was admitted for a genetic syndrome evaluation and poor weight gain. He was hypoxemic with cardiomegaly on chest X-ray prompting an echocardiogram which revealed a VSD with pulmonary vein hypoplasia and stenosis. A CT done at that time revealed ostial stenosis of the pulmonary veins. The child underwent VSD closure and removal of the pulmonary venous membrane. He did well post-operatively, but was re-admitted to the cardiac intensive care unit 1 month later due to tachypnea and work of breathing. Repeat echocardiogram and CT demonstrated recurrent stenosis, right lower pulmonary vein hypoplasia, and right upper pulmonary vein atresia. Due to the rapidly progressive stenosis despite treatment, the child was deemed not a surgical candidate and was transferred to palliative care.”

—Guillermo Moreno, MD, Head of Cardiac Intensive Care Unit, Hospital de Pediatria Professor Dr. Juan P Garrahan, Buenos Aires, Argentina

A full list of demographics and extra-cardiac anomalies are listed in Table 2. 52.6% of the children (*n* = 30) had no operations prior to their PVS repair, 40.4% had one cardiac surgery prior, 5.3% had 2 prior cardiac surgeries, and 1 patient (1.8%) had 3 cardiac surgeries previously. Table 3 shows the concurrent procedure, as applicable, done at the time of PVS repair. The median ICU stay was 91 h with interquartile range (IQR) of 46–220 h. The median ventilatory time was 38 h with IQR of 19–96 h.

Six children had no outcome listed at 30 days leaving 51 cases to analyze mortality. 78.4% of children were alive at the time of our query (*n* = 40) and 21.6% deceased. In-hospital mortality occurred in 91% of cases with 1 out of hospital mortality (9%). These outcomes are given in Table 4. Eight of the 10 in-hospital mortalities were in children less than age 1 year and 2 cases were between ages 1 and 5 years. Two children who died were premature and the remainder were >37 weeks at birth. Half of the children who died had isolated pulmonary vein repairs, 2 had TAPVR repair, 1 had TAPVR plus Glenn, 1 had PAPVR repair with mitral valvuloplasty and repair of cor tri atriatum, and 1 had repaired right ventricular outflow tract obstruction. Two of the children who died had a major infection at time of death. 

## 4. Discussion

We found multiple cases of PVS that were repaired in LMIC. We found most patients had anomalous pulmonary vein repairs, but there was a near equal number of isolated PVS cases. There were many cases with concurrent left to right shunt repairs, which is a newer physiology association with PVS [14]. Prematurity was uncommon with only 6 cases in our cohort. Infectious complications were rare and present in only 11% of the group. Mortality occurred in 11 (22%) of patients with known 30-day outcomes with 18% of that occurring in the hospital within 30 days. Most of the mortalities were in children with isolated PVS repairs, but anomalous pulmonary vein repairs made up the remainder. 1/3 of patients with an infection or prematurity died. This overall suggests more complex anatomy or patient factors could be a risk factor for post-operative mortality, although the overall numbers are quite small.

This is a relatively large cohort considering the largest published cohorts describing isolated PVS are 93 patients in the US and 58 patients in an international cohort from three high income countries [15,16]. However, our cohort is a mixture of pre and post-surgical cases and may not reflect true idiopathic PVS. Previous studies have shown prematurity, multivessel disease or bilateral disease at diagnosis, and vessel hypoplasia were risk factors mortality in idiopathic vein disease, but more specific indicators of disease severity have not yet been developed [15,17,18]. Interestingly, nearly 30% of our cohort had no additional repairs outside of PVS which is higher than other reports [19,20]. This could be a reflection of coding errors from the primary sites, but could also represent high rates of idiopathic PVS. Prematurity was uncommon in this cohort, but a good percentage of these infants died. PVS is increasingly recognized as a complication of severe bronchopulmonary dysplasia and rates of PVS may increase as prematurity survival rates increase in LMICs. Additional noncardiac structural anomalies, chromosomal anomalies or other major medical illnesses were infrequent suggesting countries are limiting repairs to children who are otherwise healthy. Infectious complications occurred at a similar rate to other reports from IQIC [12,21]. Of those who had infections, 2 died supporting the importance of preventing post-surgical infections, one of the major goals of IQIC [10,11,13]. Post-operative mortality was high at 22%, but is well within published North American mortality rates of 12–48% post-PVS repair [19,20,22]. This finding could be a selection bias as children who were overall healthier or had less severe disease pre-operatively may have been offered surgery more than children who had severe disease. It does, however, speak to the feasibility of PVS repair in LMICs in appropriately selected patients in the short term.

Despite these encouraging results, challenges in the repair of PVS in LMIC exist. A major limitation in repair of PVS is related to the relatively few centers that offer repair in each country. For instance, in Malaysia, only 4 centers offer surgical repair of TAPVR and these can be physically remote from the patient’s location. This confers transportation, financial, and emotional limitations which can be insurmountable. A sick child in a city distanced from home can lead to separation of families and loss of employment by parents which can be devastating to the family in the short and long term. Frequently, multi-disciplinary teams are convened to discuss the possible long-term outcome of the patient including response to treatment coupled with social factors to determine surgical candidacy. Notably, cost of treatment is not always prohibitive with the advent of social security and similar government programs to pay for treatment. With few centers offering treatment, there are long waiting lists, so patients accrue morbidities making them less than ideal surgical candidates. This has been, in some part, alleviated by the increasing availability of catheterization procedures to stent vertical or pulmonary veins as a palliative measure while waiting. Post-operative outcomes have also improved with the increasing availability of extracorporeal membrane oxygenator (ECMO) and inhaled nitric oxide to survive low cardiac output syndrome and pulmonary hypertension, two more common complications of PVS repair. Further, sutureless PVS repair confers improved outcomes and has made post-operative care less complex so more cases can be undertaken to improve experience and outcomes. Finally, improved echocardiographic techniques and the use of advanced cross-sectional imaging such as CT have allowed for better selection of candidates. Patients with pulmonary vein hypoplasia and atresia, higher risk anatomies, can be easily identified with these modalities and those patients can be managed palliatively. LMICs interested in growing CHD surgical programs should anticipate these balancing factors in addition to achieving satisfactory surgical results.

There are limitations to this study. IQIC’s mission is related to surgical outcomes so not all cases of PVS in each country are captured. There are likely significantly more cases of PVS diagnosed, but not offered surgical repair. Issues with improper entry of diagnosis are low due to routine auditing, but PVS was selected in some cases when pulmonary valve stenosis was evident from other notes on the case. Therefore, it is possible some cases were incorrectly entered and missed. Some cases of PVS were due to mechanical obstruction related to surgical technique which is not the pathology we aim to study. However, less than half of kids had previous cardiac surgeries and only some of those had previous pulmonary vein procedures. Thus, our findings predominantly reflect PVS due to intraluminal myofibroproliferation which was our aim. Finally, while all countries are invited to participate, most of the countries who reported PVS cases were middle income. This is likely due to middle income countries abilities to handle these cases, but is nonetheless a limitation of generalizability.

## 5. Conclusions

Overall, this study demonstrates the broad reach of PVS and shows LMIC are performing the repairs with similar success to high income countries with more resources. PVS is rare and an under-studied disease with high mortality. Additional research into risk factors for mortality and best treatment practices would allow countries with limited resources to risk stratify and offer therapy to patients with the best chances of survival. Further, large PVS databases including LMIC would increase the number of patients studied so broader understanding could be developed.

## Figures and Tables

**Table 1:** List of institutions who reported cases of PVS that were repaired.

Institution
Shanghai Children’s Medical Center (Shanghai, China)
TEDA International Cardiovascular Hospital (Tianjin China)
First Hospital of Lanzhou University (Lanzhou, Gansu Province, China)
Nhi Dong 1 (Children’s Hospital #1) (Ho Chi Minh City, Vietnam)
Vietnam National Children’s Hospital (Hanoi, Vietnam)
Tam Duc Heart Hospital (Ho Chi Minh City, Vietnam)
Institut Jantung Negara (Kuala Lumpur, Malaysia)
Amrita Institute of Medical Science (Kochi, India)
Kokilaben Dhirubhai Ambani Hospital & Medical Research Center (Mumbai, India)
Frontier Lifeline Hospital (Chennai, India)
G. Kuppuswamy Naidu Memorial Hospital (Coimbatore, India)
Fortis Child Heart Mission, Fortis Hospital, Mulund (Mumbai, India)
Mother and Child Health Institute (Belgrade, Serbia)
Hospital Garrahan (Buenos Aires, Argentina)
Hospital de Niños (Córdoba, Argentina)
Clínica Cardio VID (Medellin, Columbia)
Fundación Cardioinfantil de Bogota (Bogota, Colombia)
Fundación Valle del Lili (Cali, Colombia)
Hospital do Coração (Sao Paolo, Brazil)
Instituto do Coração (Sao Paolo, Brazil)
Dr. Carlos Alberto Studart Gomes Hospital (Fortaleza, Brazil)
Hospital de criança e Maternidade (Sao Jose do Rio Preto, Brazil)
Instituto Nacional de Pediatría Mexico City, Mexico
Hospital Nacional de Niños (San Jose, Costa Rica)

**Table 2:** Cohort demographics and extra-cardiac anomalies. The predominant age was less than 1-year and males accounted for slightly more than half of the cohort. On average, the children had normal hematocrits and saturations. 14 children carried diagnoses outside of congenital heart disease.

Variable	Percentage of Total or Median (*n* or IQR)
Age <1 year	57.9% (33)
Age 1–5 years	26.3% (15)
Age 6–14 years	15.8% (9)
% Male	56.1% (32)
Prematurity	10.5% (6)
Major non-cardiac structural anomaly	3.5% (2)
Major chromosomal anomaly	3.5% (2)
Major medical illness	7.0% (4)
Weight	6.0 kg (4.5–9.5 kg)
Hematocrit	36% (33–40%)
Systemic saturation	95% (90–99%)

**Table 3:** General cardiac surgery categories. One patient who underwent TAPVR repair also had a Glenn. Nearly one-third of patients had isolated PVS repair and slightly more than a third had anomalous pulmonary venous repair. Left to right shunts were commonly repaired. Note, some cases of anomalous pulmonary vein repairs may have had left to right shunts (i.e., ASDs) that were repaired, but not noted in the record.

Primary Procedure	Number	Percentage of Total Cohort (*n* = 57)
Isolated PVS repair	17	29.8%
Anomalous pulmonary vein repair	20	35.1%
Left to right shunt repair	15	26.3%
VSD repair	5	
ASD repair	5	
VSD and ASD repair	2	
Transitional AV Canal repair	2	
PDA ligation	1	
Pulmonary outflow tract obstruction relief	4	7%
Bidirectional Glenn	2	3.6%

**Table 4:** Survival and infectious outcomes. Infections were overall uncommon. Mortality occurred in less than one-third of cases. * 30-day status was not available for 6 patients.

Outcome	Percent or Number of Patients with Outcome
Infections	10.5% (*n* = 6)
Bacterial sepsis	4
Surgical site infection	1
Both	1
Mortality	
In hospital mortality	17.5% (*n* = 10)
30-day mortality	21.6% * (*n* = 11)

## Data Availability

Data available on request due to privacy restrictions. The data are not publicly available due to identifiable patient data present in the database.
